# Clinical significance of the reduced expression of G protein gamma 7 (*GNG7*) in oesophageal cancer

**DOI:** 10.1038/sj.bjc.6604124

**Published:** 2008-01-22

**Authors:** M Ohta, K Mimori, Y Fukuyoshi, Y Kita, K Motoyama, K Yamashita, H Ishii, H Inoue, M Mori

**Affiliations:** 1 Department of Surgery, Medical Institute of Bioregulation, Kyushu University, Beppu, Japan; 2 Division of Stem Cell Regulation/Molecular Hematopoiesis, Jichi Medical School, Center for Molecular Medicine, Tochigi, Japan

**Keywords:** cancer suppressor gene, large G protein, loss of heterozygosity, methylation, microRNA

## Abstract

We previously cloned human *G protein gamma 7* (*GNG7*) and demonstrated that it was downregulated in gastrointestinal cancer. The significance of *GNG7* expression in oesophageal cancer is unknown. TaqMan quantitative real-time PCR was performed to determine the clinical significance of *GNG7* expression in 55 cases of oesophageal cancer. Furthermore, *GNG7*-transfected oesophageal cancer cells were analysed in laboratory studies at genomic and epigenetic levels. Twenty-seven patients with low *GNG7* expression showed significantly poorer survival than did 28 patients with high expression (*P*<0.05). Tumours with low *GNG7* expression invaded deeper than those with high *GNG7* expression (*P*<0.05), both *in vivo* and *in vitro*. Eight tumours retained *GNG7* expression, and they did not show either promoter hypermethylation or loss of heterozygosity (LOH). In 38 tumours with *GNG7* suppression, 22 (57%) showed either LOH or promoter hypermethylation. In addition, *GNG7* expression was significantly associated with the presence of miR328 in oesophageal cancer cell lines, which suggests that this microRNA might be a regulator of *GNG7* expression. *GNG7* suppression represents a new prognostic indicator in cases of oesophageal cancer. *GNG7* might be suppressed by LOH and promoter hypermethylation or by microRNA.

Cell signalling mediated by small guanine nucleotide-binding proteins (G proteins) such as ras (Bos, 1989), rho (Symons, 1995; Boivin *et al*, 1996), and rac (Boivin *et al*, 1996) has been reported in various cancers. In contrast, oncogenic changes mediated by heterotrimeric large G proteins have been unknown and most analyses of large G proteins have focused on solid carcinomas. In our previous studies, we reported that large G protein gamma 7 (GNG7) was downregulated in pancreatic cancer patients (Shibata *et al*, 1998) in 70% of gastrointestinal cancers and in 94% of intrahepatic cholangiocarcinomas (Utsunomiya *et al*, 2002).

Among diverse solid carcinomas, we are presently focusing on the relationship between loss of *GNG7* expression and its clinical significance in oesophageal carcinomas. Oesophageal cancer is epithelial in origin with a high malignant potential and most patients die from it (Holscher *et al*, 1995). Thus, we sought to identify a marker for oncogenesis and progression of oesophageal cancer. In the current study, we examined the clinical significance of diminished *GNG7* expression in oesophageal cancer cases and determined whether its expression was altered at the genomic level, mRNA level, epigenetic level or the microRNA level.

In general, tumour suppressor gene function may be lost at genomic levels due to mutation, homozygous deletion, loss of heterozygosity (LOH) and/or epigenetic changes such as DNA methylation or histone deacetylation (Garinis *et al*, 2002). In addition, recent studies disclosed that expression of several genes was regulated by microRNA expression. With regard to expression of microRNA, likely molecules that could be regulated can be predicted based on information provided by a recently developed website (http://microrna.sanger.ac.uk/targets/v3/). In this study, two such microRNA molecules were examined for their regulation of *GNG7* expression in cultured cell lines.

In preliminary studies, we evaluated the expression of *GNG7* in 10 oesophageal cancer patients (Shibata *et al*, 1999). In the current study, we significantly expanded that work with additional cases and analysed clinicopathologic information to determine the precise role of *GNG7* in oesophageal cancer.

## MATERIALS AND METHODS

### Cell lines and tissues

Fifteen human oesophageal carcinoma cell lines were prepared. The TE series was provided by the Cell Resource Center for Biomedical Research Institute of Development, Aging and Cancer, Tohoku University, Japan. The KYSE series was a generous gift from Dr Shimada (Kyoto University, Kyoto, Japan). *GNG7* transfectants with pcDNA3-GNG7 (*GNG7* expression vector) and mock cell lines were established in KYSE150 according to methods described previously (Shibata *et al*, 1999). After 24 h, the cells were subcultured and selected in G418-containing medium. After 2 weeks, colonies were transferred separately to individual wells and established as transfectants. Real-time PCR (RT-PCR) and immunoblotting confirmed *GNG7* expression in the transfectants. Primary tumour tissue specimens and corresponding normal tissue controls were obtained from 55 Japanese patients who underwent surgery for oesophageal carcinoma (47 men and 8 women; median age, 61 years; range, 40–82 years). Written informed consent was obtained from these patients. Histologically, 47 tumours were classified as squamous cell carcinoma, two as adenocarcinoma and six as other types. Follow-up ranged from 3 to 4159 days.

### DNA and RNA extraction

Tissue samples were excised and immediately stored at −80°C. DNA and RNA were extracted according to methods described previously. Tumour tissues containing more than 80% tumour cells had shown H&E staining.

### TaqMan quantitative RT-PCR analysis

TaqMan RT-PCR for *GNG7* and 18s cDNA was performed using an ABI PRISM 7000 (Applied Biosystems, Tokyo, Japan; Chen, 2005), according to the manufacturer's instructions and our previous study (Shibata *et al*, 1999). The expression levels were normalised to 18s rRNA. The cases were then classified into two groups according to the median expression value (0.32), that is, a *GNG7* high-expression group (tumour/normal (T/N)>0.32; *n*=27) and a *GNG7* low-expression group (T/N<0.32: *n*=28).

### Invasion assay

The invasion assay was performed in 24-well trays using inserts with 8 *μ*m pore size (Becton Dickinson, Franklin Lakes, NJ, USA) as described previously. For invasion assays, inserts were coated with Matrigel (100 *μ*g cm^−2^) (Becton Dickinson). Oesophageal cancer cells were suspended in RPMI-1640 medium at 5 × 10^4^ ml^−1^ and added to inserts, which were transferred to wells containing RPMI-1640 medium with 10% FBS. After incubation for 6 or 24 h, cells on the lower surface of the membrane were stained for light microscopy. Five different fields were assessed (× 200). Assays were performed in triplicate.

### LOH analysis

In the following studies, we utilised 42 of 55 cases, all with informed consent. We defined abnormal *GNG7* mRNA expression in the tumour tissue as an expression that was less than 20% of that seen in normal tissue specimens. Paired normal and tumour DNA samples were analysed with two polymorphic microsatellite markers. Allelic deletions of chromosome 19p region flanking the *GNG7* gene were assessed using markers D19S1166 and D19S565. The former region is −0.6 Mb from the *GNG7* gene, while the latter is an intragene marker. All of the microsatellite markers used in this analysis were CA-repeat markers. The primer sequences were obtained from the Genome Database (D19S1166: forward: TTCCAGCCTAGGTAGCAGTG, reverse: GCAACTGAGGAAATGCATCTD; 19S565: forward: GTGATTGCACCACGGG, reverse: TCAAGTCATTGGGTTGGC). PCR was performed in 10 *μ*l reaction volumes containing 0.1 *μ*M each of FAM-labelled forward and unlabelled reverse primers, 0.19 mM dNTP mix, 1 × PCR buffer, 0.4 U AmpliTaq Gold polymerase (Applied Biosystems) and 100 ng of genomic DNA. Amplification was performed in an iCycler (Bio-Rad, Hercules, CA, USA) with an initial cycle of 95°C for 5 min, followed by 30 cycles of 95°C for 50 s, 55°C for 60 s and 72°C for 60 s, with a final extension at 72°C for 10 min. After 1 *μ*l of each PCR product was diluted with 7 *μ*l of DNase-free, RNase-free distilled water, 0.1 *μ*l of each diluted sample was added to 0.7 *μ*l of formamide and 0.3 *μ*l of ROX 400HD size standard and denatured at 95°C for 5 min before loading the samples into the Applied Biosystems 373 DNA sequencer (Applied Biosystems). Analysis of raw data and assessment of LOH were performed with GeneScan Analysis v.2.0.0 software (Applied Biosystems). Cases met LOH criteria when an allele peak signal from tumour DNA was reduced by 50% compared with the normal counterpart.

### Bisulphite treatment

We extracted genomic DNA and carried out bisulphite modification of genomic DNA as described previously (Yamashita *et al*, 2002). Briefly, 2 *μ*g of DNA in 20 *μ*l of H_2_O containing 5 *μ*g of salmon sperm DNA were denatured by incubation with 0.3 M NaOH at 50°C for 20 min. The DNA was then incubated at 70°C for 3 h in a 500-*μ*l reaction mixture containing 2.5 M sodium metabisulphite and 0.125 M hydroquinone (pH 5.0). The treated DNA was purified with the Wizard DNA purification system according to the manufacturer's instructions (Promega, Madison, WI, USA) and finally resuspended in 100 *μ*l of H_2_O after ethanol precipitation.

### 5-AZAC treatment of oesophageal cancer cell lines

To evaluate the methylation status of the promoter region of *GNG7* in oesophageal cancer, cell lines were treated with a demethylating drug, 5-AZAC, as in our previous study (Mori *et al*, 1996). We analysed *GNG7* expression by quantitative RT-PCR before and after 5-AZAC treatment in 15 oesophageal cancer cell lines.

### Methylation analysis

The assay involved sodium bisulphite modification followed by sequence and pyrosequencing to determine the methylation status of *GNG7* promoter region. DNA methylation patterns in the promoter region of the *GNG7* gene were (Bottoni *et al*, 2005) determined by sequencing bisulphite-treated DNA. We used three paired primers for the analysis. The primers were as follows: F1, 5′-TAATCTTTCCTAACTCCCACTAAAA-3′; R1, 5′-AGTAGTAGAGTAGTGGTGGTTTAAT-3′; F2, 5′-CCAAAACCAAAAAATCAATA-3′; R2, 5′-TTTTAGTGGGAGTTAGGAAAGATTA-3′; F3, 5′-CCAACCCCACAATCAAAATCCAATC-3′; and R3, 5′-TTTTTATTGATTTTTTGGTTTTGGG-3′. PCR was performed in 25-*μ*l reaction volumes containing 16.6 mM ammonium sulphate, 67 mM (pH 8.8) Trizma, 6.7 mM MgCl_2_, 10 mM 2-mercaptoethanol, 10 mM DMSO, 1.25 mM dNTP, 1.6 *μ*M of each primer, 2.5 U of platinum Taq (Perkin-Elmer, Foster City, CA, USA), and 50 ng of bisulphite-treated DNA. PCR cycling conditions (Touchdown PCR) were as follows: 5 min at 95°C, two cycles of 60 s denaturation at 94°C, 60 s at 66°C annealing temperature, a 120 s extension at 72°C, two cycles of 60 s at 94°C, 60 s at 64, 62, 60, 58, and 56°C, 120 s at 72°C, 30 cycles of 60 s at 94°C, 60 s at 54°C, 120 s at 72°C, and a final extension step of 7 min at 72°C. To verify the methylation status, DNA bands of each transcript were excised from the gel, purified using the QIAquick gel extraction kit (Qiagen, Valencia, CA, USA) and sequenced on the Applied Biosystems Prism 377 DNA sequencing system.

### Pyrosequencing-based typing

We chose a region located in CpG islands of *GNG7* for PSQ analysis to detect methylation status. PCR primers were designed to be fully complementary to the deaminated strand (forward: 5′-GAAAGTGGAAATTGGGGATAAAT-3′, reverse: 5′-biotin label-CCTCCAAACCTTAATAAAAATCAAA-3′). For this region, PCR was performed in 30 *μ*l reaction volumes containing 16.6 mM ammonium sulphate, 67 mM (pH 8.8) Trizma, 6.7 mM MgCl_2_, 10 mM 2-mercaptoethanol, 10 mM DMSO, 1.25 mM dNTP, 1.6 *μ*M of each forward and reverse (biotinylated) primer, 25 ng of bisulphite-converted DNA as template, and 2.5 U of platinum Taq (Perkin-Elmer). The PCR programme consisted of a denaturing step of 4 min at 95°C followed by 50 cycles of 30 s at 95°C, 30 s at 60°C, 60 s at 72°C, with a final extension for 5 min at 72°C. The amplification cycle was repeated 50 times to ensure complete consumption of the biotinylated primers to avoid background signals during pyrosequencing. Within the PCR region, we designed three sequencing primers (5′-AAGTGGAAATTGGGGATAAA-3′, 5′-GGTTAATAGGAAGGAAAGAA-3′, 5′-GGTTAGGTTTYGTATT-3′) to cover four CpG arrangements. A PCR product (25 *μ*l) was used for each sequencing reaction. Purification with streptavidin sepharose beads and codenaturation of the biotinylated PCR products and the sequencing primer (15 pmol per reaction) were conducted following the PSQ sample preparation guide using a filter plate device. After completion of primer annealing, 500 ng of SSB was added to the sequencing reaction mix. Sequencing was performed on the PSQ 96 MA system with SQA reagent kit according to the manufacturer's instructions. Pyrosequence data were quantified and background corrected using PSQ 96 MA version 2.0.2 software (Pyrosequencing AB, Stockholm, Sweden). Tumour tissues were defined as methylation-positive when a methylation allele peak signal from tumour DNA was 10% elevated compared with the normal counterpart.

### Regulation of *GNG7* expression and the presence of microRNA

Total RNA was extracted from 16 oesophageal cancer cell lines: TE6, TE8, TE10, TE12, TE14, TE15, KYSE110, KYSE140, KYSE180, KYSE220, KYSE270, KYSE450, KYSE500, KYSE700, KSE1 and KSE2. When the cell density reached 75% confluency, RNA was extracted using the Trizol® total RNA isolation reagent (Gibco BRL, Life Technologies, Gaithersburg, MD, USA) as per the manufacturer's protocol. Total RNA was isolated from frozen tissues after disruption with an Ultra Turrax T25 homogeniser using Trizol. RNA concentrations were quantified using a NanoDrop Spectrophotometer (NanoDrop Technologies, Wilmington, Delaware, USA). cDNA was synthesised from total RNA using gene-specific primers according to the TaqMan MicroRNA Assays protocol (PE Applied Biosystems, Foster City, CA, USA). Reverse transcriptase reactions contained 10 ng of RNA, 50 nM stem-loop RT primer, 1 × RT buffer, 0.25 mM each of dNTPs, 3.33 U *μ*l^−1^ MultiScribe reverse transcriptase and 0.25 U *μ*l^−1^ RNase Inhibitor (all purchased from cDNA Archive kit of Applied Biosystems). The 7.5 *μ*l reactions were incubated in a Bio-Rad i-Cycler in a 96-well plate for 30 min at 16°C, 30 min at 42°C, 5 min at 85°C, and then held at 4°C. Real-time PCR for microRNA was performed using an Applied Biosystems 7500 Real-Time PCR System. The 10 *μ*l PCR included 0.67 *μ*l RT products, 1 × TaqMan Universal PCR master mix and 1 *μ*l primers and probe mix according to the TaqMan MicroRNA Assays protocol (PE Applied Biosystems). The reactions were incubated in 96-well optical plates at 95°C for 10 min, followed by 40 cycles of 95°C for 15 s and 60°C for 10 min. Those data were determined using the default as the fractional cycle number at which fluorescence passed the fixed threshold. According to the website (http://microrna.sanger.ac.uk/targets/v3/), two kinds of microRNA, miR15a (Hoglund *et al*, 1998) and miR328, were chosen as candidates to regulate *GNG7* expression. On the basis of the PCR for miR15a and miR328, the amplified products were present in 10 and 16 out of 16 cell lines, respectively.

### Statistical analysis

Fisher's exact test and Student's *t*-test were used for statistical analysis; *P*-values <0.05 were regarded as statistically significant.

## RESULTS

### Expression of *GNG7* mRNA in oesophageal tumours, normal tissues and cell lines

**Figure 1:**
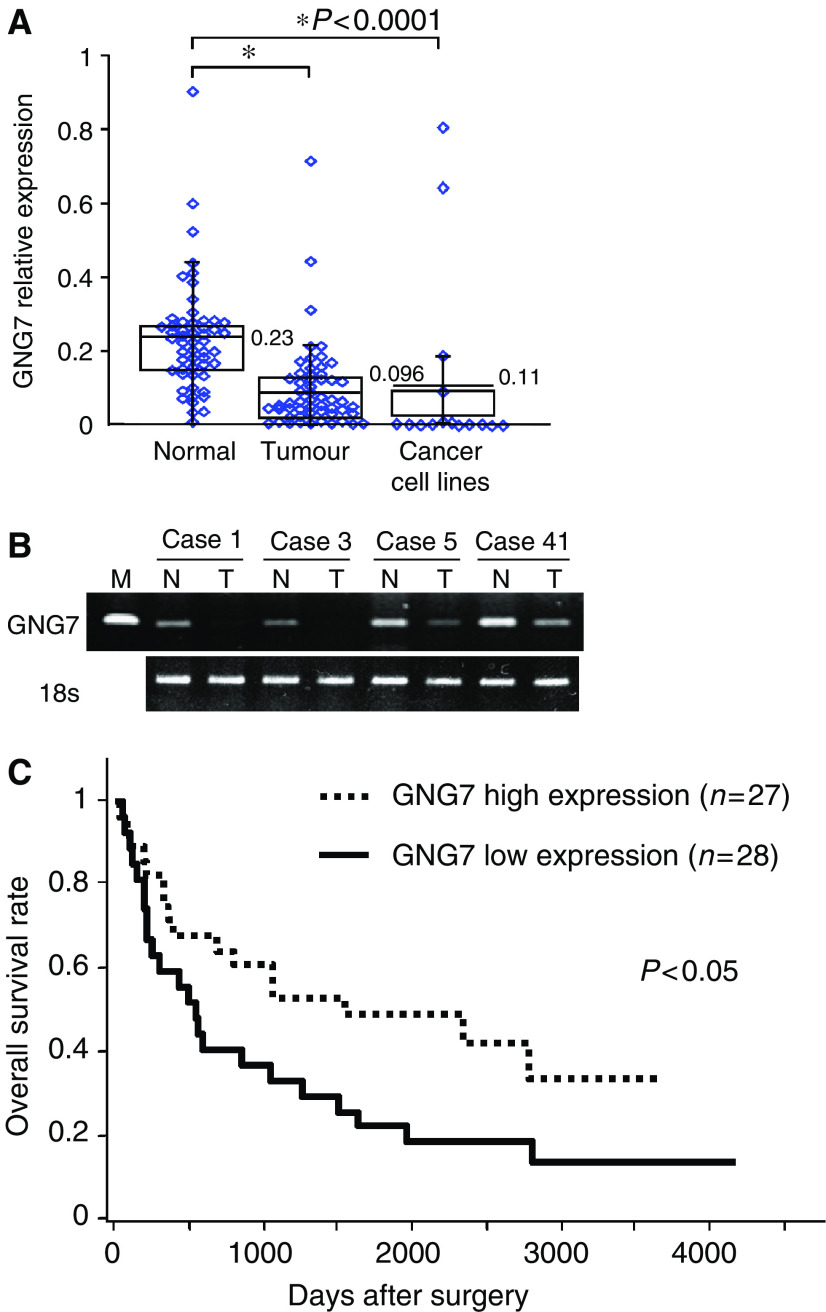
Expression of *GNG7* mRNA and patient survival. (**A**) Expression of *GNG7* mRNA in tumour samples, normal tissues and cell lines. *GNG7* mRNA expression was determined in oesophageal cancer tissues and corresponding normal tissues obtained from 55 Japanese patients who underwent surgery for oesophageal carcinoma. Figure also shows *GNG7* expression in 15 cell lines established from oesophageal cancer tissue. Expression ratios were calculated by dividing the amplified *GNG7* expression level by the expression of 18s ribosomal RNA. Tumour samples (relative expression: 0.096) expressed *GNG7* at significantly lower levels than did normal samples (relative expression: 0.23) (*P*<0.0001). (**B**) Electrophoresed amplified products of *GNG7* and GAPDH in tumour and the corresponding normal tissue from four representative oesophageal cancer cases. (**C**) Survival curves of 55 cases of oesophageal cancer in relation to *GNG7* expression. Oesophageal cancer patients were divided into two groups relative to the median expression value (0.32), yielding a *GNG7* high-expression group (T/N>0.32; *n*=27) and a *GNG7* low-expression group (T/N<0.32; *n*=28).

As shown in Figure 1, tumour samples (relative expression: 0.096) expressed *GNG7* at significantly lower levels than did normal samples (relative expression: 0.23) (*P*<0.0001). Interestingly, in 45 of 55 oesophageal cancer tissue specimens (82% of total), the level of *GNG7* suppression was at least 80% compared to the level of corresponding normal tissue specimens. In oesophageal tumour cell lines, the average expression level of *GNG7* was quite low (relative expression: 0.11), similar to results obtained with clinical tumour samples. It is worth describing that the *GNG7* expression in cancer cell lines was low in comparison with samples from cancer tissues. In contrast to the cancer tissues that contained normal stromal cells, the 15 cancer cell lines were not contaminated with normal cells. Therefore, we speculated that clinical samples with stromal cells showed much higher *GNG7* expression than the cancer cell lines. Furthermore, we divided 55 oesophageal cancer cases into two groups based on the median relative expression value for *GNG7* (T/N=0.32). Thus, 27 cases were above the median and considered in the high *GNG7* expression group, while 28 were placed in the low *GNG7* expression group.

### Correlation of clinicopathologic factors and the expression of *GNG7* in oesophageal cancer

Clinicopathologic analysis of the high- and low-expression groups showed that low *GNG7* expression was significantly associated with greater depth of tumour invasion (Table 1, *P*<0.05). The survival curve of 55 patients in relation to *GNG7* expression status is shown in Figure 1C. The patients with low *GNG7* expression showed a significantly poorer survival than did those with high expression (*P*<0.05). In contrast, no significant differences were observed in *GNG7* gene expression status when patients were categorised by age, gender, histological type, lymph node metastasis, lymph vessel permeation and venous permeation. Considering clinical application, even though 12 cases with tumour were restricted within the oesophageal wall, 9 cases (75%) were classified into the poor prognosis group. Although multivariate analysis did not disclose the statistical significance of *GNG7* in predicting prognosis, several cases of tumour restricted in the oesophageal wall and with lower expression of *GNG7* were useful in predicting poorer prognosis than the depth of tumour invasion.

### Invasion assay

**Figure 2:**
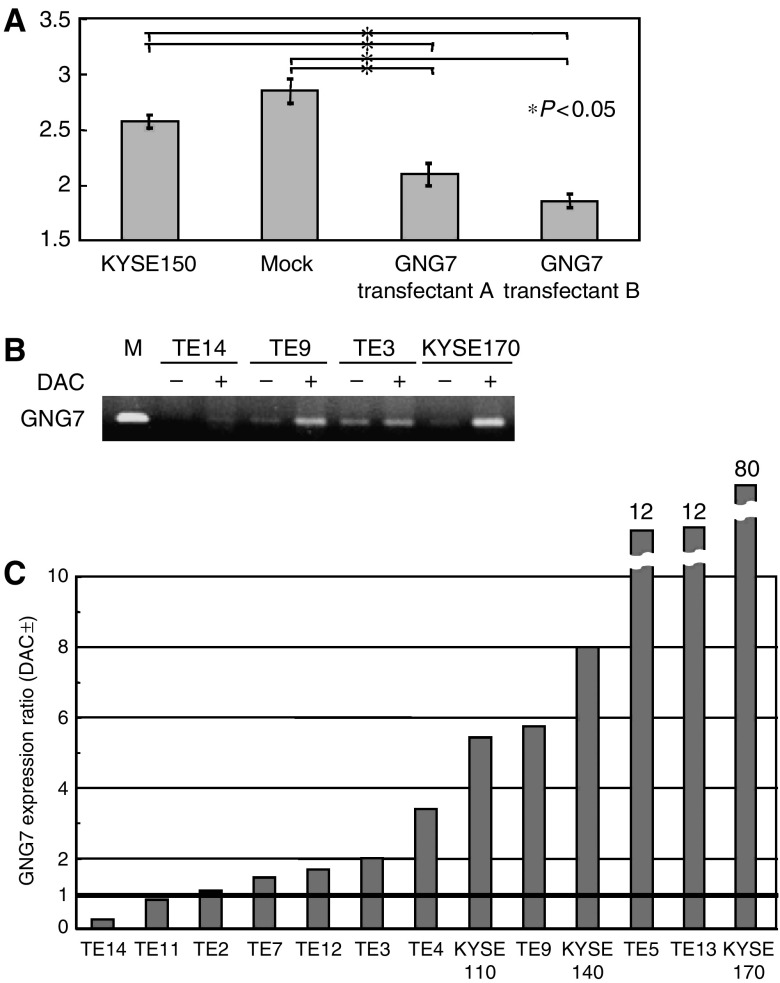
Biologic characteristics of *GNG7*-transfected oesophageal cancer cells. (**A**) Invasion assay for *GNG7* transfectants. After incubation for 6 or 24 h, the number of cells on the lower surface of the membrane was counted in five different fields (× 200). Clone A and clone B *GNG7* transfectants were compared with the parental cell line KY150 and the mock cell line. *GNG7* transfectants A (average intensity=2.08) and B (average intensity=1.87) were significantly less invasive than parental cells (average intensity=2.55) and mock cells (average intensity=2.82) (Figure 3, *P*<0.05). (**B**) Reactivation of *GNG7* expression by 5-AZAC treatment. Analysis of four representative oesophageal cancer cell lines. Data indicate that *GNG7* expression may be regulated by methylation. (**C**) Increased expression of *GNG7* after 5-AZAC treatment in 15 oesophageal cancer cell lines. The vertical axis indicates the calculated ratio of *GNG7* expression with and without 5-AZAC treatment.

Since the expression of *GNG7* was associated with the depth of tumour invasion, the transwell invasion assay was performed to determine whether invasive activity was directly mediated by expression of *GNG7* in oesophageal cancer cell lines. We compared *GNG7* transfectants with the parental cell line KYSE150 and a mock cell line. High expressers of *GNG7* (transfectant A, average intensity=2.08 and transfectant B, average intensity=1.87) were significantly less invasive than KYSE150 (average intensity=2.55) and the mock cell line (average intensity=2.82) (Figure 2A, *P*<0.05). These *in vitro* results were concordant with clinicopathologic data and support the hypothesis that *GNG7* modulates tumour cell invasiveness.

### LOH analysis at 19p13.3

**Figure 3:**
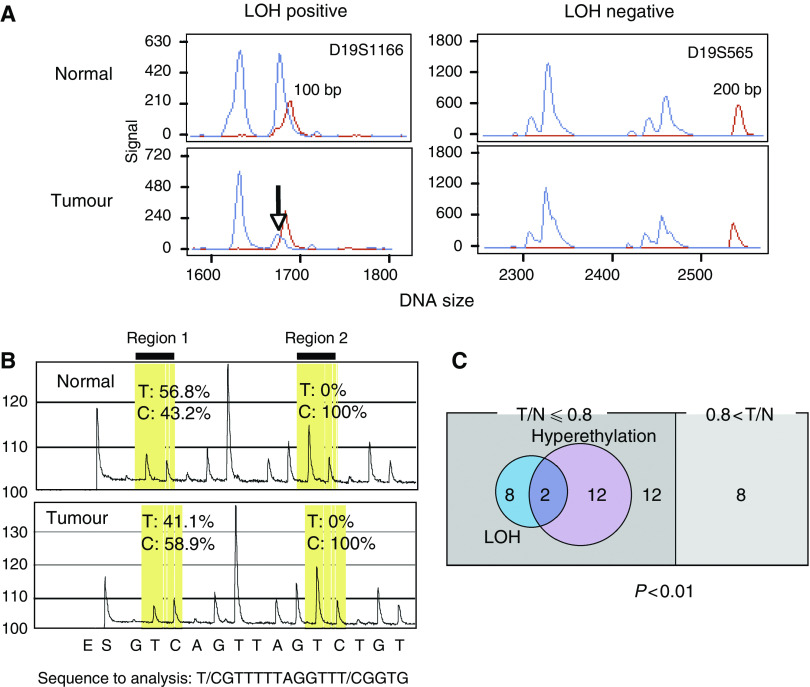
Genetic alterations of the *GNG7*-coding region. (**A**) Two representative oesophageal cancer cases that were positive (left) or negative (right) for LOH. (**B**) Results of pyrosequencing analysis in a CG-rich region. Region #2 did not show any alterations by bisulphite treatment. However, region #1 showed that nucleotide C was increased in tumour tissues relative to corresponding normal tissue, and nucleotide T was decreased in tumour compared to corresponding normal tissue. This trial focused on the CG site with cancer-specific CG arrangements. (**C**) Classification of 42 cases of oesophageal cancer according to LOH status at *GNG7* region and/or promoter hypermethylation. No genetic and/or epigenetic alterations were detected in eight cases (100%) of oesophageal cancer that lacked suppression of *GNG7* (T/N<0.8) expression. In contrast, LOH and/or hypermethylation were found in 22 out of 34 cases (65%) with diminished *GNG7* expression.

We first examined the LOH in the *GNG7* region (Figure 3A). Loss of heterozygosity in the *GNG7* region at 19p13.3 was examined in 42 oesophageal cancer cases using two microsatellite markers. The frequencies of LOH for each microsatellite marker in informative cases were 9 out of 32 (28%) and 3 out of 29 (10%) for D19S1166 and D19S565, respectively. Loss of heterozygosity was detected in 10 out of 40 (25%) tumours that were informative for at least one of the two markers at the 19p13.3 region. We also examined the relationship between LOH and the suppression ratio of *GNG7* mRNA (Table 2). All 10 LOH-positive cases (100%) had suppressed expression of *GNG7* (T/N<0.8). There was a significant correlation between the presence of LOH and the suppression ratio of *GNG7* mRNA (*P*<0.01).

### Reactivation of *GNG7* expression by 5-AZAC treatment

In 9 out of 15 (60%) oesophageal cell lines (TE3, TE4, TE5, TE8, TE9, TE13, KYSE110, KYSE140 and KYSE170), *GNG7* expression was restored by 5-AZAC treatment (Figure 2B and C). Thus, promoter hypermethylation may have contributed to *GNG7* suppression.

### Methylation analysis of the *GNG7* promoter region in clinical oesophageal cancer

Methylation of the *GNG7* promoter region was assessed in 42 oesophageal cancer patients (paired tumour and normal DNA). To evaluate DNA methylation patterns in the promoter region of the *GNG7* gene, we carried out bisulphite sequencing in five paired cancer and normal tissue specimens. These five cases showed suppressed *GNG7* expression in tumour tissue compared with normal tissue. There are 41 CG arrangements in the promoter region of *GNG7*. Although most of these CG arrangements were methylated in both tumour and normal DNA samples, there were some unmethylated CG arrangements in normal DNA samples compared with corresponding tumour DNA samples (data not shown). On the basis of these findings, we utilised quantitative methylation analysis (pyrosequencing analysis) (Figure 3B).

In pyrosequencing analysis, the frequencies of hypermethylated CG arrangements in oesophageal cancer cases were 10 out of 42 (24%) and 6 out of 42 (14%) at regions 1 and 2, respectively. Overall, hypermethylation was detected in 14 out of 42 (33%) tumours in one of the two CG regions at the *GNG7* promoter. We also examined the relationship between the presence of hypermethylation and *GNG7* expression status (Table 2). All 14 hypermethylation-positive cases (100%) had suppressed *GNG7* expression (T/N<0.8). There was a significant correlation between the presence of hypermethylation and *GNG7* expression status (*P*<0.01). These data suggest that promoter hypermethylation may contribute to the mechanism of *GNG7* suppression.

### Correlations among hypermethylation, LOH and RNA expression

We analysed the correlation between *GNG7* gene expression and various mechanisms of gene suppression. That is, we determined if there were correlations between the levels of mRNA expression and promoter hypermethylation status and/or LOH at *GNG7* region (Figure 3C). Interestingly, there was a significant correlation between expression and the pattern of hypermethylation and LOH at *GNG7* (*P*<0.01). In the eight cases of normal *GNG7* expression (0.8<T/N), there were no cases with either promoter hypermethylation or LOH. In the 34 cases with *GNG7* suppression (T/N⩽0.8), there were two cases with both LOH and promoter hypermethylation. There were eight patients positive for LOH and 12 that showed promoter hypermethylation. Thus, in 34 patients showing *GNG7* suppression (T/N⩽0.8), 22 cases (65%) could be explained by LOH and/or promoter hypermethylation.

### Significant association between expression of *GNG7* and microRNAs

**Figure 4:**
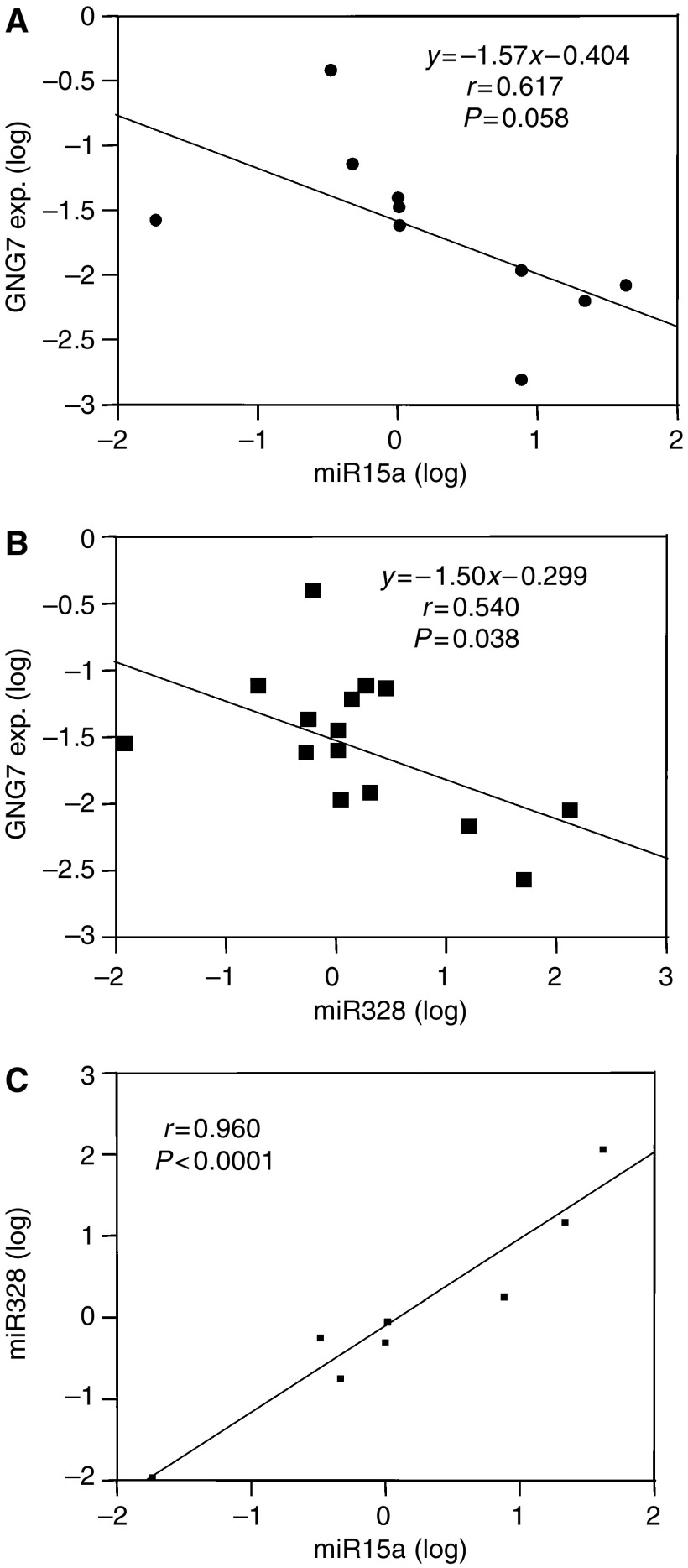
Relationship between *GNG7* expression and two predicted microRNAs, miR15a and miR328. (**A**) miR15a tended to be associated with *GNG7* expression in 10 cell lines (TE6, TE8, TE10, TE12, KYSE110, KYSE450, KYSE700, KSE1 and KSE2) (*r*=0.617, *P*=0.058). (**B**) There was a significant association between miR328 and *GNG7* expression in TE6, TE8, TE10, TE12, TE15, KYSE110, KYSE140, KYSE180, KYSE220, KYSE270, KYSE450, KYSE500, KYSE700, KSE1 and KSE2. (**C**) Expression of miR328 and miR15a was highly correlated (*r*=0.96, *P*<0.001).

In Figure 4, *GNG7* expression was inversely and significantly associated with miR328 expression (*P*<0.05) in 16 oesophageal cancer cell lines. The expression of miR15a also tended to be inversely associated with the level of *GNG7* expression, but the correlation did not reach significance. Therefore, these experiments showed that *GNG7* could be repressed by miR328.

## DISCUSSION

In this study, we demonstrated that expression of *GNG7* was frequently (82% of cases) and strongly suppressed in oesophageal cancer tissues compared with normal oesophageal tissues. We also found that *GNG7* suppression was associated with invasiveness and poorer prognosis. The latter may be due to the former because we found that re-expression of the *GNG7* gene in oesophageal cancer cell lines reduced invasiveness.

We analysed the mechanism by which *GNG7* expression was reduced in oesophageal cancer. Loss of chromosome 19p13.3, which includes the *GNG7* locus, has been observed frequently in several cancers (Ritland *et al*, 1995; Hoglund *et al*, 1998; Wang *et al*, 1999; Kato *et al*, 2004; Miyai *et al*, 2004; Yang *et al*, 2004). Furthermore, STK11, the Peutz–Jeghers gene, is also located on the telomeric region of chromosome 19p13.3 (Hemminki *et al*, 1998; Jenne *et al*, 1998). In our oesophageal cancer study, LOH in *GNG7* region (19p13.3) was detected in 10 out of 40 patients (25%). In fact, LOH was significantly correlated with reduced *GNG7* expression (*P*<0.01). Thus, *GNG7* suppression was partially related to LOH in oesophageal cancer. However, an epigenetic alteration may also explain the reduced expression of *GNG7* in oesophageal cancer.

In oesophageal cancer, it is known that p16, FHIT and VHL are inactivated by promoter hypermethylation (Wong *et al*, 1997; Kuroki *et al*, 2003). We showed that expression of *GNG7* in oesophageal cancer cell lines could be restored by treatment with the demethylating drug 5-AZAC. This suggests that in a model system, *GNG7* promoter hypermethylation might be responsible for silencing *GNG7* expression. Extending that observation, we also found *GNG7* promoter hypermethylation in oesophageal cancer tissues. Taken together, these data suggest that promoter hypermethylation may be intimately associated with *GNG7* suppression.

Interestingly, this study demonstrated that *GNG7* promoter hypermethylation occurred in advanced stage tumours as well as in early tumours. Specifically, it was recognised in 3 out of 12 tumours restricted within the oesophageal wall. These three included one tumour of submucosal invasion and two tumours of proper muscular invasion. Therefore, based on these findings, we suggest that the hypermethylation of the *GNG7* promoter might be an early event in the progression of oesophageal cancer. Interestingly, bisulphite sequencing and pyrosequencing analysis of the promoter region of *GNG7* revealed that it was moderately methylated in non-cancerous oesophageal tissue (data not shown). The presence of hypermethylation in corresponding non-cancerous tissue may indicate a precancerous condition in the surrounding non-cancerous area of the tumour. In fact, Eads *et al* (2001) reported a similar phenomenon: moderate promoter methylation in non-cancerous tissue and increased methylation in cancer tissue.

Finally, we demonstrated that microRNAs may modulate *GNG7* translation. The presence of the predicted microRNAs was significantly associated with *GNG7* expression. Thus, expression of *GNG7* may be partially controlled by microRNAs. At this point, there are insufficient data to determine the extent to which genomic alteration, hetero- and homo-allelic loss, epigenetic alterations (as methylation) and microRNAs contribute to altered *GNG7* expression. Esquela-Kerscher and Slack (2006) reported that microRNA is an abundant class of small, non-protein-coding RNAs that function as negative gene regulators. Our data confirm that finding. Chen (2005) reported that microRNA itself can be an oncogene or a suppressor gene. The present study is the first to demonstrate the role of miR328 in the regulation of a cancer suppressor gene, *GNG7*. In accordance with the finding of cancer cell lines that seven oesophageal cancer cell lines with more than two times higher reactivation by DAC treatment were not associated with the level of miR328 expression; we therefore speculated that there should be no relationship between miR328 status and the epigenetic and genetic alterations in determining the *GNG7* mRNA expression in clinical oesophageal cancers. In addition, we found that the presence of miR328 and miR15a was closely associated with one another (Figure 4C, *r*=0.96, *P*<0.001). Therefore, a common upstream factor may control both miR328 and miR15a to regulate *GNG7* expression.

In summary, we demonstrated that *GNG7* was frequently and strongly suppressed in oesophageal cancer. *GNG7* suppression was associated with greater invasiveness of cancer cells in the oesophageal wall and poorer prognosis. Its expression was suppressed by LOH at the *GNG7* locus and epigenetic hypermethylation of the *GNG7* promoter, and regulated by at least two microRNAs. Further analysis of the signal cascade through which *GNG7* acts is needed to understand its precise molecular function. We anticipate that analysis of *GNG7* expression should provide an important prognostic indicator in the near future.

## Figures and Tables

**Table 1:** Relationship between clinicopathologic variables and GNG7 expression in oesophageal cancer patients

	**GNG7 expression**		
	**High**	**Low**	***P*-value**
*Age (years)*			0.209
<62	14	18	
>62	14	9	
			
*Sex*			0.476
Male	23	24	
Female	5	3	
			
*Histology* a			0.576
Well	6	7	
Mod	14	9	
Poor	4	7	
Other	4	4	
			
*Depth* b			<0.05
Within	3	9	
Beyond	25	18	
			
*Lymph node metastasis*			0.10
Negative	8	3	
Positive	20	24	
			
*Lymph vessel permeation*			0.451
Negative	11	8	
Positive	17	19	
			
*Venous permeation*			0.134
Negative	16	10	
Positive	12	17	

GNG7=G protein gamma 7.

a Well=well-differentiated squamous cell carcinoma; mod=moderately differentiated squamous cell carcinoma; poor=poorly differentiated squamous cell carcinoma; other four cases are mucinous adenocarcinoma.

b Within and beyond the oesophageal wall.

**Table 2:** Relationship between the loss of GNG7 expression and the genetic and/or epigenetic alteration in its loci

	**LOH**			**Hypermethylation**	
	−	+	**NI**	−	+
*GNG7 expression*					
T/N<0.8	24	10	0	20	14
0.8<T/N	6	0	2	8	0
*P*-value			0.0043		0.0065
					

GNG7=G protein gamma 7; LOH=loss of heterozygosity; T/N=expression ratio of GNG7 between tumour and the corresponding normal tissues.
